# The Construct Validity of the Life‐Space Assessment in the Canadian Longitudinal Study on Aging (CLSA)

**DOI:** 10.1155/jare/4755246

**Published:** 2026-07-15

**Authors:** Selina Malouka, Marla Beauchamp, Julie Richardson, Bruce Newbold, Ayse Kuspinar

**Affiliations:** ^1^ School of Rehabilitation Science, McMaster University, Hamilton, Ontario, Canada, mcmaster.ca; ^2^ Department of Health Research Methods, Evidence, and Impact, McMaster University, Hamilton, Ontario, Canada, mcmaster.ca; ^3^ Faculty of Science, McMaster University, Hamilton, Ontario, Canada, mcmaster.ca

**Keywords:** CLSA, community-dwelling adults, life-space assessment, mobility, validity

## Abstract

**Background:**

Life‐space mobility refers to the way individuals maneuver themselves in their home and community and is commonly assessed using the Life‐SpaceAssessment (LSA). Before using the LSA, it is important to ensure that it demonstrates validity in the population being used, as culture, built environment, and modes of transportation in different geographical regions can influence mobility. Therefore, the purpose of this study was to assess the convergent, divergent, and known‐groups validity of the LSA in community‐dwelling Canadian middle‐aged and older adults using data from the Canadian Longitudinal Study on Aging (CLSA).

**Methods:**

Spearman’s correlation coefficients were calculated for all measures of convergent (hypothesis: *r* > 0.5) and divergent (hypothesis: *r* < 0.3) validity. Known‐groups validity was assessed by determining if the LSA can differentiate between people with chronic diseases and a history of falls using measures of clinical significance (i.e., LSA score change of 5 or more points).

**Results:**

The sample (*n* = 24,577) had a median LSA score of 86.0. Correlations between the LSA and comparator measures of convergent validity (*r* = 0.10–0.29) did not meet the hypothesis; however, measures of divergent validity (*r* = 0.15–0.17) met the hypothesis. For known‐groups validity, the LSA can be used to differentiate between those with (median score = 84; interquartile range [IQR] = 72–100) and without (median score = 90; IQR = 74–100) a history of falls among the whole sample. Further, it can be used to differentiate between those with and without chronic conditions, among males and females aged 75+ years.

**Conclusion:**

The LSA can be used to differentiate between known groups. However, there is limited evidence of its convergent validity as its relationship with other measures of actual mobility, perceived mobility, and locomotor capacity for mobility was weak. Future studies need to assess the convergent validity of the LSA against different comparator measures before its use among community‐dwelling Canadians.

## 1. Introduction

Conceptualizing mobility in a holistic manner requires understanding the way individuals maneuver themselves in their home and community. Rapidly emerging concepts used to describe community mobility are life‐spaces and life‐space mobility. Life‐spaces are the areas individuals visit in their everyday life (e.g., their home, areas outside the home, and their neighborhood), and life‐space mobility refers to the extent of movement across various life‐spaces, regardless of the method of mobility [1]. The most commonly used measure of life‐space mobility is the Life‐SpaceAssessment (LSA), which is a patient‐reported outcome measure [2–4].

There are different types of mobility measures (e.g., performance‐based and self‐reported measures), and a mismatch is often reported between the level of functioning that individuals report as being *capable* of and what they *actually* achieve in their daily life [5–7]. The discordance is further exemplified by the often weak relationships between performance‐based and self‐reported measures of functioning, suggesting that they are not measuring the same construct [8]. The unified framework for mobility measurement in older adults was proposed to illustrate this apparent mismatch and describe the three “facets” of mobility [9]: (1) perceived mobility (self‐report measures of what a person *can* do), (2) locomotor capacity for mobility (what a person *could* do as measured by a test), and (3) actual mobility (what a person *actually does* in daily life) [9]. Actual mobility can be measured using self‐report measures (e.g., the LSA) or direct measures (e.g., accelerometry) and is influenced by situational and environmental factors in day‐to‐day life. According to this framework, the LSA captures the facet of actual mobility.

Before adopting a measure in clinical or research settings, it is important to ensure that the scores produced demonstrate validity in the population of interest. LSA scores have not been validated among community‐dwelling middle‐aged and older adults in Canada. Country‐specific validation is needed, considering that culture (e.g., social relationships and educational/occupational opportunities) [10], built environments [11, 12], and modes of transportation [13] can influence mobility. The purpose of this study was to assess the construct validity (convergent, divergent, and known‐groups) of the LSA in community‐dwelling Canadian middle‐aged and older adults using data from the Canadian Longitudinal Study on Aging (CLSA) [14].

## 2. Methods

### 2.1. Study Design

The CLSA [14] is a large, randomly selected, national sample of 50,000 individuals aged 45–85 years across Canada followed for a minimum of 20 years. Individuals residing within 25–50 km of the following centers were included to represent the four regions of Canada: the Pacific Coast (Victoria [University of Victoria], Vancouver, and Surrey [University of British Columbia and Simon Fraser University]), the Prairies (Calgary [University of Calgary] and Winnipeg [University of Manitoba]), Central Canada (Hamilton [McMaster University], Ottawa [University of Ottawa], Montréal [McGill University], and Sherbrooke [Université de Sherbrooke]), and the Atlantic Region (Halifax [Dalhousie University] and St. John’s [Memorial University of Newfoundland]). All participants provided a set of information on demographic, social, physical/clinical, psychological, economic, and health service utilization relevant to health and aging. Over 30,000 CLSA participants are asked to provide more in‐depth information through physical examinations and biological specimen collection. A cross‐sectional analysis of the CLSA Comprehensive Baseline Dataset [14] was conducted, which included ∼30,000 participants 45–85 years old, excluding participants who had missing data on the LSA, an injury over the last 12 months that limited normal activities, surgery in the last three months, chemotherapy in the past four weeks, and were receiving dialysis treatment, as these factors could temporarily affect LSA scores. Following the exclusions, the sample size was 24,577.

### 2.2. Main Outcome Measure

The LSA [4] asks whether an individual visited different spaces over the last 4 weeks with “no” scoring 0 and “yes” scoring the following points for each level: 1 = rooms outside of the bedroom, 2 = yard/outdoor area, 3 = neighborhood, 4 = other neighborhoods, and 5 = outside their city/town. Participants report the frequency of their visits to each level (i.e., a score of 1 = < 1/week, 2 = 1–3 times/week, 3 = 4–6 times/week, and 4 = daily) and if assistance was needed (i.e., a score of 1 = personal assistance, 1.5 = assistive devices, and 2 = no assistance). Scores for each level are multiplied, then summed, yielding a total score from 0 to 120, with higher scores indicating higher life‐space mobility. A clinically meaningful change among community‐dwelling older adults, developed using anchor‐based estimates, is five or more points [4, 15].

### 2.3. Outcome Measures to Assess Convergent Validity

For convergent validity, the LSA was compared against other measures of mobility, as per the unified framework for mobility measurement (Table 1). Where available, psychometric properties are reported.

**TABLE 1:** The CLSA comparator measures mapped onto each facet of mobility defined by the unified framework for mobility measurement in older persons [9].

Facets of mobility	Perceived mobility	Locomotor capacity for mobility	Actual mobility
CLSA variable	Older Americans Resources and Services (OARS) Multidimensional Assessment Scale	Timed Up and Go (TUG) test	Life‐Space Assessment (LSA)
		4‐M Walk Test (4MWT)	Physical Activity Scale for the Elderly (PASE)
		Single Leg Stance (SLS)	Driving frequency
		Chair Rise (CR) Test	Frequency of public transportation use

*The Physical Activity Scale for the Elderly (PASE*) assesses physical activity over the last 7 days, including leisure, household, and work/volunteer activities [16]. Higher scores indicate greater levels of physical activity. Among community‐dwelling adults, the PASE showed good test–retest reliability (*r* = 0.75, 95% CI: 0.69–0.80) [16] and moderate to strong correlations with the 6‐minute walk test (*r* = 0.68) [17].

*Frequency of public transportation use* is assessed using the question “In the past month, how frequently did you take public transit?” Responses ranged from daily to less than once a month. Higher scores indicate lower usage. Only participants who use public transportation were included.

*Frequency of driving* is assessed using the question “How frequently do you drive?” with response options ranging from daily to less than once a month. Higher scores indicate lower driving frequencies.

*The Older Americans Resources and Services (OARS) Multidimensional Assessment Scale* [18, 19]: An adapted version of this scale was used to assess activities of daily living (ADL) and instrumental activities of daily living (IADL) in the CLSA, using seven items each. The CLSA derived variable was used, which classified a participant’s capacity ranging from 1 (no functional impairment) to 5 (total impairment). The original OARS scale has been shown to have evidence of construct validity in community‐dwelling older adults [20].

*The 4-MeterWalk Test (4MWT)* measures the time taken for an individual to walk 4 meters at their usual pace, in seconds. Higher scores indicate a lower level of functioning. It demonstrated excellent test–retest reliability, with an intraclass correlation coefficient (ICC) = 0.96 in healthy older adults [21] and evidence of convergent validity with a moderate association with the 6‐minute walk test (*r* = 0.59 for men, *r* = 0.49 for women) in community‐dwelling older adults [22].

*The Timed Up and Go (TUG) test* measures the time taken, in seconds, for an individual to stand up from a chair, walk 3 meters, turn around, walk back to the chair, and sit down. Higher scores indicate a lower level of functioning. In healthy adults or adults without disability, the TUG demonstrated excellent test–retest reliability (ICC = 0.96–0.97) [23] and good concurrent validity with the 6‐minute walk test (*r* = 0.72) [23].

*The Chair Rise (CR) test* measures the time taken, in seconds, for a participant to stand up and sit down five times as quickly as possible, with no rest. Higher scores indicate a lower level of functioning. It has been shown to have good reliability (ICC = 0.71) [24] and excellent correlation with the TUG (*r = *0.92) and gait speed test (*r = *0.94) in community‐dwelling older adults [25].

*The Single Leg Stance (SLS)* test measures the time, in seconds, an individual can maintain balance standing on one leg. The best attained time on a single leg (i.e., right versus left) was used. Higher scores (with a maximum of 60 s) indicate better balance. The SLS has been shown to have excellent intra‐ and inter‐rater reliability (ICC = 0.93 – 0.99) among community‐dwelling older adults [26].

### 2.4. Outcome Measures to Assess Divergent Validity

Divergent validity was assessed by comparing the LSA against measures capturing unrelated constructs.

*The Satisfaction with Life Scale (SWLS)* [27] is a five‐item measure of satisfaction with life, with seven response options of different levels of agreement/disagreement. Total scores range between 5 and 35, with higher scores indicating higher levels of satisfaction.

*The Outcomes Study (MOS) Social Support Survey (SSS)* [28, 29] is a measure of social support availability consisting of 19 items generating four subscales of functional support: tangible social support, affection, positive social interaction, and emotional or informational support. An overall functional support index is calculated and transformed to a scale ranging from 0 to 100, with higher scores indicating greater levels of support.

### 2.5. Outcome Measures to Assess Known Groups Validity

*Chronic Disease*: Participant responses were dichotomized to include people who have at least one chronic disease and those who had none. Some chronic conditions commonly reported by the participants include allergies, hypertension, diabetes, mood disorders, asthma, and osteoarthritis of the knee.

*History of Falls*: Individuals were asked if they had one or more falls in the past 12 months (i.e., yes or no) and were categorized as fallers and nonfallers.

### 2.6. Data Analysis

Means and standard deviations (SD) were presented for continuous variables consistent with a normal distribution; counts and percentages were presented for categorical data. For data not consistent with a normal distribution, median and interquartile values were presented. For convergent and divergent validity, Spearman’s correlations were used. For known‐groups validity, median LSA scores were compared. Known‐groups validity was assessed by determining if the LSA scores can differentiate between known groups by a clinically significant difference of five or more points [4, 15]. Statistical significance was not assessed as recommended by COSMIN guidelines [30], and because of the large sample size, trivial differences are likely to show statistical significance despite not being clinically meaningful. Analyses were then stratified by age and sex. STATA statistical software was used.

Hypotheses were guided by COSMIN guidelines [30, 31]. For convergent validity, we hypothesized high correlations (> 0.5) between the LSA and comparator measures of the same facet of mobility and moderate correlations (0.3–0.5) between the LSA and comparator measures of related but dissimilar constructs (i.e., other facets of mobility). For divergent validity, we hypothesized that there would be low correlations (< 0.3) between the LSA and measures of unrelated constructs. For known‐groups validity, we hypothesized that the LSA would be able to differentiate between different known groups clinically.

## 3. Results

The comprehensive CLSA dataset (*n* = 30,097) was used with the following exclusions: individuals with missing values on the LSA (*n* = 53), surgeries in the last three months (*n* = 1243), injuries over the last 12 months that limited normal activities (*n* = 4362), chemotherapy treatments in the last four weeks (*n* = 95), and current dialysis treatment (*n* = 14). Table 2 presents the characteristics and outcome measures for the whole sample (*n* = 24,577), males (*n* = 2083), and females (*n* = 12,494). The mean age of the sample was 63.2 years (SD = 10.3), with the majority having a postsecondary degree/diploma (77.0%), at least one chronic disease (91.3%), no history of falls (90.2%), and living in an urban location (90.6%).

**TABLE 2:** Sample characteristics for the whole sample (*n* = 24,577), males, and females.

Sample characteristics				
Characteristic		*N* (%)		
		Whole sample	Males	Females
Sex	Males	12,083 (49.2)	—	—
	Females	12,494 (50.8)	—	—

Age (years)	45–54	6055 (24.6)	2909 (24.1)	3146 (25.2)
	55–64	7955 (32.4)	3848 (31.9)	4107 (32.9)
	65–74	6107 (24.9)	3058 (25.3)	3049 (24.4)
	75+	4460 (18.2)	2268 (18.8)	2192 (17.5)

Education	< Secondary school graduation	1400 (5.7)	615 (5.1)	785 (6.3)
	Secondary school graduation	2403 (9.8)	1033 (8.6)	1370 (11.0)
	Some postsecondary education	1802 (7.3)	869 (7.2)	933 (7.5)
	Postsecondary degree/diploma	18,929 (77.0)	9540 (79.0)	9389 (75.2)

Geographical location	Urban	22,273 (90.6)	11,001 (91.1)	11,272 (90.2)
	Rural	2304 (9.4)	1082 (9.0)	1222 (9.8)

Chronic disease	Present	22,435 (91.3)	10,794 (90.2)	11,641 (93.7)
	Absent	1957 (8.0)	1177 (9.8)	780 (6.3)

History of falls	Yes	2306 (9.8)	957 (8.3)	1349 (11.3)
	No	21,209 (90.2)	10,601 (91.7)	10,608 (88.7)

The median LSA score for the sample was 86.0, suggesting that the sample had no restrictions in life‐space mobility, and can move beyond their neighborhood independently [32]. When stratified by sex, median LSA scores for females (84.0) were lower than males (90.0) by a clinically important difference. Measures of physical performance (i.e., capacity for locomotor mobility) were comparable between males and females, except for the SLS where males had higher scores (58.2) than females (48.8) (Table S1). In terms of actual mobility, males had higher PASE scores (140.0) than females (120.6); however, the frequency of driving and public transportation use was comparable between males and females. Measures of perceived mobility (i.e., OARS Multidimensional Assessment Scale) and measures unrelated to mobility (i.e., SWLS and MOS‐SSS) were comparable between males and females (Table S1).

Table 3 presents Spearman’s correlation coefficients for convergent validity. Among the whole sample, the highest correlation was between the LSA and driving frequency (a measure of actual mobility) at *r* = −0.29, with the PASE (a measure of actual mobility) at *r* = 0.28 and with the OARS Multidimensional Assessment Scale (a measure of perceived mobility) at *r* = 0.24. For locomotor capacity for mobility, the LSA correlated with the SLS at *r* = 0.24, 4‐MWT at *r* = −0.22, TUG at *r* = ‐0.21, and CR at *r* = −0.10. When stratified by sex and age, correlations generally increased with age and were higher for females but remained small and still did not meet the hypotheses (Table 3 and Tables S2 and S3).

**TABLE 3:** Spearman’s (*r*) correlations for comparator measures for convergent validity of the LSA for the whole sample and stratified by sex.

Measure	Correlation *r* (95% confidence interval) with the LSA		
	Whole sample	Males	Females
Driving frequency	−0.29 (−0.30 to −0.27), *n* = 21,146	−0.26 (−0.28 to −0.25), *n* = 10,704	−0.29 (−0.31 to −0.27), *n* = 10,442
Physical activity scale for the elderly (PASE)	0.28 (0.27–0.29), *n* = 23,258	0.26 (0.24–0.28), *n* = 11,437	0.28 (0.26–0.30), *n* = 11,821
The Older Americans Resources and Services (OARS) Multidimensional Assessment Scale	−0.24 (−0.26 to −0.23), *n* = 24,501	−0.21 (−0.23 to −0.19), *n* = 12,054	−0.25 (−0.27 to −0.24), *n* = 12,447
Single leg stance (SLS)	0.24 (0.22–0.25), *n* = 23,349	0.19 (0.17–0.21) *n* = 11,535	0.28 (0.26–0.30) *n* = 11,814
4‐m walk test (4MWT)	−0.22 (−0.23 to −0.20), *n* = 24,277	−0.16 (−0.18 to −0.14), *n* = 11,942	−0.26 (−0.28 to −0.25), *n* = 12,335
Timed up and go (TUG) test	−0.21 (−0.22 to −0.19), *n* = 24,259	−0.15 (−0.17 to −0.14), *n* = 11,927	−0.27 (−0.28 to −0.25), *n* = 12,332
Chair rise (CR)	−0.10 (−0.12 to −0.09), *n* = 23,583	−0.07 (−0.09 to −0.05), *n* = 11,615	−0.13 (−0.15 to −0.12), *n* = 11,968
Public transportation frequency	0.10 (0.07–0.12), *n* = 6815	0.10 (0.06–0.13), *n* = 3361	0.09 (0.05–0.12), *n* = 3454

*Note:* All reported correlations are statistically significant at the < 0.0001 level. 95% confidence intervals are calculated with bootstrap reps = 1000.

For divergent validity, the LSA was compared with unrelated measures, such as the SWLS (*r* = 0.17) and the MOS‐SSS (*r* = 0.15). Among the whole sample, the correlations remained below the 0.3 threshold, supporting the hypothesis for divergent validity (Table 4). The correlations were also < 0.30 when the analyses were repeated for each sex separately (Tables S2 and S3).

**TABLE 4:** Spearman’s (*r*) correlations for comparator measures for divergent validity for the LSA for the whole sample and stratified by sex.

Measure	Correlation *r* (95% confidence interval) with the LSA		
	Whole sample	Males	Females
Satisfaction with life scale (SWLS)	0.17 (0.16–0.18), *n* = 24,301	0.14 (0.12–0.16), *n* = 11,954	0.20 (0.18–0.21), *n* = 12,347
Medical outcomes study (MOS) social support survey (SSS)	0.15 (0.14–0.16), *n* = 24,577	0.13 (0.12–0.15), *n* = 12,083	0.16 (0.15–0.18), *n* = 12,494

*Note:* All reported correlations are statistically significant at the < 0.0001 level. 95% confidence intervals are calculated with bootstrap reps = 1000.

Table 5 presents the results for known‐groups validity in terms of clinical significance (i.e., 5 points or more [4, 15]). The LSA could differentiate between those with and without a history of falls. When stratified by age and sex, the LSA was able to differentiate between those with and without a history of falls among males aged 65–74 years and between those with and without the presence of chronic disease among males aged 75+ years. Among females, the LSA was able to differentiate between those with and without a chronic disease among those aged 75+ years and among the entire female sample.

**TABLE 5:** Results for known‐groups validity, stratified by sex and age.

Sex	Age (years)	Median (25th to 75th *Q*) LSA scores			
		Chronic disease		History of falls	
		Present	Absent	Present	Absent
Males	45–54	92 (82–100)	92 (84–100)	92 (80–100)	92 (82–102)
	55–64	90 (80–100)	92 (82–100)	90 (74–100)	90 (80–100)
	65–74	90 (74–100)	90 (80–100)	84 (72–100)^∗^	90 (76–100)^∗^
	75+	82 (72–92)^∗^	90 (80–100)^∗^	80 (69–92)	82 (72–92)
	All Males	90 (76–100)	92 (82–100)	90 (72–100)	90 (78–100)

Females	45–54	90 (80–100)	90 (81–100)	90 (80–100)	90 (80–100)
	55–64	86 (74–100)	90 (80–100)	86 (74–100)	86 (74–100)
	65–74	82 (68–92)	86 (74–100)	80 (68–92)	82 (70–92)
	75+	74 (62–86)^∗^	81 (68–95)^∗^	74 (60–84)	74 (64–86)
	All Females	82 (72–96)^∗^	90 (80–100)^∗^	82 (72–96)	84 (72–96)

Whole sample		86 (74–100)	90 (82–100)	84 (72–100)^∗^	90 (74–100)^∗^

^∗^Clinical significance of 5 points or more.

## 4. Discussion

This study assessed the construct validity of the LSA in community‐dwelling Canadian middle‐aged and older adults. Convergent validity results suggested that the LSA scores had low and comparable correlations with all facets of mobility, even when stratified by age and sex. Despite not meeting our hypothesis for convergent validity, the LSA demonstrated slightly higher associations with measures of actual and perceived mobility than measures of locomotor capacity for mobility. In our study, driving frequency was most highly correlated with the LSA. Perhaps, given that the LSA captures a holistic view of community mobility, it could be capturing aspects of the construct of driving frequency. Shah and colleagues investigated the relationship between driving status and life‐space mobility among 571 community‐dwelling older adults [33]. Their results showed that, even after adjusting for age, sex, and education, having a driver’s license was associated with a decreased likelihood of limitations in life‐space mobility (hazard ratio = 0.39; 95%, CI = 0.29–0.54) over an average follow‐up period of 4.3 years [33]. Moreover, driving cessation has also been linked to significant declines in LSA scores among 669 community‐dwelling older adults after 6‐year follow‐up [34].

One explanation for the low correlations observed between the LSA and measures of the same facet could be due to differences in the constructs being measured. For example, while both the PASE and LSA are measures of actual mobility, the PASE is a measure of physical activity, while the LSA is a measure of life‐space mobility. When considering other measures of actual mobility, such as step count measured by an accelerometer, Spearman’s rank correlation with the LSA was 0.41 in a sample of community‐dwelling older adults aged 75–90 years old living in Finland (*n* = 174) [35]. The relatively higher correlation observed in the literature could be due to differences in sample demographics; however, step count is also another indicator of physical activity levels, not accounting for other forms of mobility (e.g., driving).

With regard to locomotor capacity for mobility, a review by Kuspinar and colleagues demonstrated that the correlations between the LSA and performance‐based measures ranged from 0.37 to 0.60 [36]. The higher correlations obtained in the literature could be due to the differences in comparator measures used, different sample sizes, and the sample characteristics (e.g., our current sample had higher LSA scores on average). Also, our sample is predominantly highly functioning so the low variability in test scores could result in low correlations overall. Lastly, the use of categorical variables for some comparator measures (e.g., driving frequency) could have led to information getting lost, thus contributing to the low correlations observed.

In terms of divergent validity, the hypotheses were met in that all comparator measures had correlation values below 0.3 with the LSA, suggesting that they are measuring different constructs. The LSA met our hypothesis for known‐groups validity by reflecting a clinically meaningful difference between those with and without a history of falls in the whole sample. When stratified by sex and age, the LSA scores were able to reflect differences between those with and without a history of falls among males aged 65–74 years old. Further, LSA scores were able to reflect differences between those with and without chronic conditions, among females, as well as among males and females aged 75+ years. However, it is important to note that while median LSA scores differed between groups, their IQR overlapped. This suggests that LSA scores could reflect differences between groups but may not be useful on an individual level. It is also important to note that results may vary depending on the cut‐off value that is used for clinical significance, and future studies should determine the clinically important difference of LSA scores in this population for use in future known‐groups validation studies. Our results for known‐group validity are consistent with previous studies that suggest that older adults (70+ years) with a chronic disease often have an increased risk of mobility decline [37]. Specifically, having one chronic disease increases this risk by 1.4‐fold, and having two or more diseases increases the risk by 2.3 times [37]. Moreover, LSA scores have been shown to be lower across certain chronic conditions, such as dementia [38], stroke [39], undernutrition [40], obesity [39], and the presence of depressive symptoms [39, 41]. Similarly, previous studies highlighted that individuals with a history of falls have displayed a decline in LSA scores after 6 months [42].

Overall, these findings highlight the mixed nature of the current literature on the correlations between the LSA and other mobility measures. Several explanations for the mixed literature should be explored; specifically, careful consideration should be given to the comparator measures chosen when assessing the validity of the LSA. On one hand, the results could be interpreted as a lack of evidence of the validity of the LSA in the current population. On the other hand, it could be indicative of limitations of the comparator measures chosen in that they do not measure the same construct (e.g., physical activity versus community mobility), or rather the same *facet* of mobility (i.e., perceived, actual, and locomotor capacity for mobility), in the same manner that the LSA does. The LSA is intended to encompass multiple forms of mobility (e.g., walking, driving, and public transportation use) and is intended to reflect the construct of *community* mobility, which is influenced by a host of psychosocial and financial factors. Perhaps, no current measure of mobility is designed to capture the same construct as the LSA, and future research should explore the associations between the LSA and other measures of mobility. One example of an emerging measure of community mobility that can be compared against the LSA could be Global Positioning System (GPS) data.

With regard to strengths and limitations, much of the literature to date on convergent and known‐groups validity of the LSA has been limited to convenience samples across narrow age groups, other countries, such as Finland or Sweden, or to specific clinical populations [43–46]. Strengths of the current study include offering a more thorough assessment of the LSA using a large population‐based dataset that provides information from various measures and across a wider age range in Canada. Further, to our knowledge, this is the first study to assess validity guided by a theoretical model to distinguish between the different facets of mobility. Given the CLSA’s exclusion criteria, the results of this study may not be applicable to all individuals and geographical locations in Canada.

## 5. Conclusion

The LSA did not meet the hypotheses for convergent validity; however, the strongest associations were found between the LSA and measures of actual and perceived mobility. For known‐groups validity, the LSA can be used to differentiate between those with and without a history of falls among the whole sample, those with and without a history of falls among males aged 65–74 years old, and those with and without chronic conditions, among males and females aged 75+ years, when using the clinically important difference of 5 or more points. As it stands, the current study does not provide evidence that the LSA correlates with measures of actual mobility, perceived mobility, and locomotor capacity for mobility, likely due to a lack of existing comparator measures of the same construct. Before using the LSA, future studies should explore the convergent validity of the LSA using different comparator measures of *actual* mobility, or perhaps a combination of existing measures, to provide more confidence in the interpretation of its scores.

## Funding

This work was supported by the Canadian Institutes of Health Research (CIHR) Funding Reference Number AC9‐187269.

## Disclosure

The opinions expressed in this manuscript are the author’s own and do not reflect the views of the Canadian Longitudinal Study on Aging (CLSA).

## Conflicts of Interest

The authors declare no conflicts of interest.

## Supporting Information

Additional supporting information can be found online in the Supporting Information section.

## Supporting information


**Supporting Information** Table S1. Sample performance on outcome measures for the whole sample (*n* = 24,577), males and females. Table S2. Spearman’s correlations for comparator measures and the LSA for females, stratified by age. Table S3. Spearman’s correlations for comparator measures and the LSA for males, stratified by age.

## Data Availability

Data are available from the Canadian Longitudinal Study on Aging (CLSA) (https://www.clsa-elcv.ca/) for researchers who meet the criteria for access to de‐identified CLSA data.
