# Supplementary material for: The Construct Validity of the Life‐Space Assessment in the Canadian Longitudinal Study on Aging (CLSA)
Source: J Aging Res. 2026 Jul 15;2026:4755246. doi: 10.1155/jare/4755246 (PMC13370320; doi:10.1155/jare/4755246)
Supplement: Supplementary file 1 — Supporting Information Table S1. Sample performance on outcome measures for the whole sample (n = 24,577), males and females. Table S2. Spearman’s correlations for comparator measures and the LSA for females, stratified by age. Table S3. Spearman’s correlations for comparator measures and the LSA for males, stratified by age. [file JARE-2026-4755246-s001.docx]

**SUPPLEMENTARY MATERIAL**

**Table S1.** Sample performance on outcome measures for the whole sample (*n* = 24,577), males and females

| Measure | *Outcome Measures* | | |
| --- | --- | --- | --- |
|  | Median (25^th^ – 75^th^ Q) | | |
|  | Whole Sample | Males | Females |
| *Life Space Assessment (LSA)* | 86 (74.0-100.0) | 90.0 (78.0-100.0) | 84.0 (72.0-96.0) |
| *Timed Up and Go (TUG) test (s)* | 9.2 (8.2-10.4) | 9.3 (8.3–10.5) | 9.2 (8.1-10.4) |
| *4-Meter Walk Test (4MWT) (s)* | 4.1 (3.7-4.7) | 4.1 (3.6-4.6) | 4.2 (3.7-4.8) |
| *Single Leg Stance (SLS) (s)* | 53.0 (13.4-60.0) | 58.2 (14.5-60.0) | 48.8 (12.3-60.0) |
| *Chair Rise (CR) (s)* | 12.9 (10.9-15.3) | 12.9 (10.8-15.2) | 13.0 (10.9-15.5) |
| *Physical Activity Scale for the Elderly (PASE)* | 130.1 (87.0-181.2) | 140.0 (95.0-193.6) | 120.6 (79.9-169.3) |
| *Satisfaction with Life Scale (SWLS)* | 30.0 (25.0-33.0) | 30.0 (25.0-33.0) | 29.0 (24.0-33.0) |
| *Medical Outcomes Study (MOS) Social Support Survey (SSS)* | 85.5 (72.4-96.1) | 86.8 (72.4-97.4) | 85.5 (72.4-96.1) |
|  | N (%) | | |
| The Older Americans Resources and Services (OARS) Multidimensional Assessment scale |  |  |  |
| 1= No functional impairment | 22,260 (90.9) | 11,390 (94.5) | 10,870 (87.3) |
| 2= Mild functional impairment | 1,978 (8.1) | 549 (4.6) | 1,429 (11.5) |
| 3= Moderate functional  impairment | 208 (0.9) | 95.0 (0.8) | 113.0 (0.9) |
| 4= Severe functional impairment | 40.0 (0.2) | 13.0 (0.1) | 27.0 (0.2) |
| 5= Total functional impairment | 15.0 (0.1) | 7.0 (0.1) | 8.0 (0.1) |
| *Public Transportation Frequency* |  |  |  |
| Daily | 455 (6.7) | 212 (6.3) | 243 (7.0) |
| 4-6 times per week | 875 (12.8) | 408 (12.1) | 467 (13.5) |
| 2-3 times per week | 1,003 (14.7) | 501 (14.9) | 502 (14.5) |
| Once a week | 957 (14.0) | 469 (14.0) | 488 (14.1) |
| Less than once a week but more  than once a month | 2,118 (31.1) | 1,075 (32.0) | 1,043 (30.2) |
| Less than once a month | 1,407 (20.7) | 696 (20.7) | 711 (20.6) |
| *Driving Frequency* |  |  |  |
| Daily | 13,930 (65.9) | 7,668 (71.6) | 6,262 (60.0) |
| 4-6 times per week | 3,969 (18.8) | 1,728 (16.1) | 2,241 (21.5) |
| 2-3 times per week | 1,984 (9.4) | 843 (7.9) | 1,141 (10.9) |
| Once a week | 392 (1.9) | 140 (1.3) | 252 (2.4) |
| Less than once a week but more  than once a month | 224 (1.1) | 90 (0.8) | 134 (1.3) |
| Less than once a month | 239 (1.1) | 98 (0.9) | 141 (1.4) |
| Not at all | 408 (1.9) | 137 (1.3) | 271 (2.6) |

*s=* seconds

**Table S2.** Spearman’s correlations for comparator measures and the LSA for females, stratified by age

|  | *r* (*95% Confidence Interval*) | | | |
| --- | --- | --- | --- | --- |
| Comparator Measure | 45-54 years | 55-64 years | 65-74 years | 75+ years |
| PASE | 0.13(0.10 to 0.16)  *n* = 2,962 | 0.21(0.18 to 0.24) *n*=3,923 | 0.23(0.19 to 0.26) *n*=2,900 | 0.26(0.22 to 0.30)  *n*= 2,036 |
| OARS Multidimensional Assessment | -0.16 (-0.20 to -0.13)  *n*=3,139 | -0.20(-0.23 to -0.16)  *n*=4,091 | -0.20(-0.23 to -0.16)  *n*=3,038 | -0.30(-0.35 to -0.26) *n*= 2,179 |
| TUG | -0.07(-0.11 to -0.04)  *n*=3,128 | -0.17(-0.20 to -0.14)  *n*=4,056 | -0.20(-0.24 to -0.17)  *n*=3,005 | -0.30(-0.34 to -0.26) *n*= 2,143 |
| SWLS | 0.18(0.15 to 0.22) *n*=3,120 | 0.20(0.17 to 0.23) *n*=4,078 | 0.21(0.18 to 0.25) *n*=3,012 | 0.18(0.14 to 0.22)  *n*= 2,137 |
| CR | -0.04(-0.07 to 0.00) ^*^ *n*=3,098 | -0.06(-0.09 to -0.03)  *n*=3,981 | -0.06(-0.10 to -0.02)  *n*=2,926 | -0.12(-0.16 to -.07) *n*= 1,963 |
| 4MWT | -0.10(-0.13 to -0.07)  *n*=3,129 | -0.17(-0.20 to -0.14)  *n*=4,057 | -0.20(-0.24 to -0.17)  *n*=3,004 | -0.31(-0.35 to -0.27) *n*= 2,145 |
| MOS-SSS | 0.15(0.12 to 0.19) *n*=3,146 | 0.15 (0.12 to 0.18) *n*=4,107 | 0.14(0.11 to 0.18) *n*=3,049 | 0.11(0.06 to 0.15)  *n*= 2,192 |
| SLS | 0.12(0.08 to 0.16) *n*=3,102 | 0.19(0.16 to 0.22)  *n*=3,974 | 0.19(0.15 to 0.22) *n*=2,887 | 0.22(0.18 to 0.27)  *n*= 1,851 |
| Public Transportation Frequency | 0.07(0.00 to 0.13) *n*=939 | 0.07(0.02 to 0.13) *n*=1,178 | 0.16(0.09 to 0.23) *n*=779 | 0.04(-0.04 to 0.12) *n*=558^*^ |
| Driving Frequency | -0.20(-0.23 to -0.16)  *n*=2,823 | -0.30(-0.31 to -0.24)  *n*=3,637 | -0.30(-0.29 to -0.21) *n*=2,443 | -0.33(-0.37 to -0.28) *n*=1,539 |

PASE = Physical Activity Scale for the Elderly; OARS = The Older Americans Resources and Services (OARS) Multidimensional Assessment; TUG = Timed Up and Go test; SWLS = Satisfaction with Life Scale; CR = Chair Rise test; 4MWT = 4 Meter Walk Test; MOS-SSS = Medical Outcomes Study Social Support Survey; SLS = Single Leg Stance.

All reported correlations are statistically significant at the <0.05 level; ^*^ = not significant at the <0.05 level.

95% confidence intervals are calculated with bootstrap reps = 1,000

**Table S3.** Spearman’s correlations for comparator measures and the LSA for males, stratified by age

|  | *r* (*95% Confidence Interval*) | | | |
| --- | --- | --- | --- | --- |
| Comparator Measure | 45-54 years | 55-64 years | 65-74 years | 75+ years |
| PASE | 0.17(0.13 to 0.21)  *n*=2,755 | 0.21(0.17 to 0.24)  *n*=3,674 | 0.22(0.19 to 0.26) *n*=2,919 | 0.23(0.18 to 0.27) *n*=2,089 |
| OARS | -0.14(-0.18 to -0.10) (n=2,907) | -0.18(-0.21 to -0.15) *n*=3,842 | -0.20(-0.23 to -0.15) *n*=3,048 | -0.26(-0.30 to -0.22)  *n*=2,257 |
| TUG | -0.06(-0.09 to -0.02)  *n*=2,889 | -0.08(-0.11 to -0.05) *n*=3,800 | -0.10(-0.14 to -0.07) *n*=3,010 | -0.17(-0.21 to -0.12)  *n*=2,228 |
| SWLS | 0.18(0.14 to 0.21)  *n*=2,892 | 0.16(0.13 to 0.20)  *n*=3,818 | 0.13(0.09 to 0.16) *n*=3,012 | 0.17(0.13 to 0.21) *n*=2,232 |
| CR | -0.01(-0.05 to 0.02)  *n*=2,860 | -0.03(-0.06 to 0.00)^*^ *n*=3,733 | -0.02(-0.05 to 0.02)^*^ *n*=2,945 | -0.05(-0.09 to -0.00) *n*=2,077 |
| 4MWT | -0.06(-0.10 to -0.028)  *n*=2,891 | -0.08(-0.12 to -0.05)  *n*=3,804 | -0.11(-0.14 to -0.07) *n*=3,011 | -0.20(-0.24 to -0.15)  *n*=2,236 |
| MOS-SSS | 0.15(0.11 to 0.18)  *n*=2,909 | 0.16(0.13 to 0.19)  *n*=3,848 | 0.12(0.08 to 0.15) *n*=3,058 | 0.08(0.04 to 0.13)  *n*=2,268 |
| SLS | 0.09(0.05 to 0.13)  *n*=2,864 | 0.11(0.08 to 0.14) *n*=3,725 | 0.12(0.08 to 0.15) *n*=2,928 | 0.10(0.06 to 0.15)  *n*=2,018 |
| Public Transportation Frequency | 0.12(0.06 to 0.18)  *n*=1,008 | 0.11(0.05 to 0.17) *n*=1,117 | 0.10(0.02 to 0.16)  *n*=800 | 0.13(0.04 to 0.21)  *n*=436 |
| Driving Frequency | -0.21(-0.25 to -0.18) *n*=2,655 | -0.26(-0.29 to -0.22) *n*=3,480 | -0.29(-0.33 to -0.25) *n*=2,689 | -0.27(-0.31 to -0.23)  *n*=1,880 |

PASE = Physical Activity Scale for the Elderly; OARS = The Older Americans Resources and Services (OARS) Multidimensional Assessment; TUG = Timed Up and Go test; SWLS = Satisfaction with Life Scale; CR = Chair Rise test; 4MWT = 4 Meter Walk Test; MOS-SSS = Medical Outcomes Study Social Support Survey; SLS = Single Leg Stance.

All reported correlations are statistically significant at the <0.05 level; ^*^ = not significant at the <0.05 level.

95% confidence intervals are calculated with bootstrap reps = 1,000
